# Perceptions on the use of medicines, vaccines, and alternative medicine during pregnancy and breastfeeding: Population-based survey in Catalonia, Spain

**DOI:** 10.18332/ejm/225498

**Published:** 2026-07-24

**Authors:** Berta Munné Barellas, Àurea Cartanyà-Hueso, Guim Arbona, Cristina Vedia-Urgell, Cristina Martínez-Bueno, Rosa Morros, Maria Giner-Soriano

**Affiliations:** 1 Institut de Recerca en Atenció Primària (IDIAPJGol), Barcelona, Spain; 2 Departament de Farmacologia, Universitat Autònoma de Barcelona, Barcelona, Spain; 3 Unitat de Farmàcia. Gerència Barcelonès Nord i Maresme. Institut Català de la Salut, Badalona, Spain; 4 Servei d’Atenció a la Salut Sexual i Reproductiva (ASSIR), Direcció Assistencial d’Atenció Primària, Institut Català de la Salut, Barcelona, Spain; 5 Sexual and Reproductive Health Care Research Group (GRASSIR), Barcelona, Spain; 6 Facultat d’Infermeria, Universitat de Barcelona, Barcelona, Spain

**Keywords:** pregnancy, breastfeeding, medication use, vaccination, complementary and alternative medicine

## Abstract

**INTRODUCTION:**

Evidence on the use, safety, and decision-making related to medication, vaccination, and complementary and alternative medicine during pregnancy and breastfeeding, remains limited. This uncertainty may influence women’s health behaviors and perceptions during these stages. The aim of this study is to identify the needs related to the use of medication, vaccination, and alternative medicine during pregnancy and breastfeeding in Catalonia.

**METHODS:**

A descriptive cross-sectional survey was conducted among 889 pregnant and recently postpartum women attending public Sexual and Reproductive Health Care Services in Catalonia (2024–2025). The questionnaire assessed use, opinions, information sources, and decision-making regarding medication, vaccination, and alternative therapies during pregnancy and breastfeeding.

**RESULTS:**

More than half of the participants reported health conditions requiring medication during pregnancy. Most women (84.6%) relied on healthcare professionals’ prescriptions and recommendations, and about half felt safe and confident when using medication. Vaccination during pregnancy was widely accepted, with over 80% agreeing with it. Alternative therapy use was limited, with osteopathy being the most common. During breastfeeding, medication use remained cautious and largely guided by professional advice.

**CONCLUSIONS:**

Pregnant and breastfeeding women generally adopt a cautious approach to medication and rely heavily on healthcare professionals for decision-making. Trust and shared decision-making play a key role in adherence and perceived safety, highlighting the importance of clear, evidence-based guidance to support maternal and infant health.

## INTRODUCTION

There is limited evidence regarding the use, adherence, and safety of medication, vaccination, and alternative medicine such as vitamins and supplements, herbs, homeopathy, and acupuncture during pregnancy and breastfeeding. The potential risks and ethical considerations of pregnancy and lactation in research^1,2^, along with androcentrism, have resulted in a lack of studies related to women’s health processes^3^.

The use of medication for various symptoms and chronic and acute conditions during pregnancy is common and, in some cases, necessary to prevent the worsening of pre-existing diseases and to minimize negative effects during pregnancy, childbirth, and on fetal health^4^. Although many women choose to take medication during pregnancy and lactation, decision-making can be complex due to the limited evidence on the safety of certain drugs^5^. At the same time, from a gender perspective, it is important to acknowledge the effects of medicalization processes on women’s health – particularly during pregnancy, childbirth, and breastfeeding^6^ – and how these may influence women’s perceptions of such products during these stages.

Regarding vaccination, certain vaccines have been shown to be safe and are recommended at specific times during pregnancy (e.g. MMR, influenza, pertussis, and COVID-19)^7^. Some of the vaccines that are considered safe and recommended still show low coverage rates during pregnancy, as uncertainties and doubts often arise when deciding whether to get vaccinated^8,9^. For this reason, shared decision-making with healthcare professionals (HCPs) is crucial to improve use and adherence, as well as to ensure informed decisions regarding these products – an approach that has been shown to enhance the overall experience of pregnancy^10^.

Women are the main users of complementary and alternative medicine, which is also commonly used during pregnancy and breastfeeding. Due to concerns about the possible teratogenic effects of many conventional medications, some women may choose what they perceive as more ‘natural’ alternatives^11^. Nevertheless, there may also be hesitation about using herbal products during pregnancy or lactation due to fears about potential effects^12^. Therefore, it is essential to understand the use of such products during these stages.

It is also important to identify the sources of information women rely on (e.g. the internet, pre- and postnatal groups, healthcare professionals, etc.) to inform themselves about pregnancy, childbirth, and breastfeeding in relation to these three categories of products, and to address the concerns they may have regarding their child’s well-being^13^. At the same time, from a gender perspective, it is necessary again to consider the effects of the medicalization and technologization of pregnancy, childbirth, and breastfeeding, to move beyond the dichotomy between the desire for the child’s well-being and women’s self-determination over their own bodies^14-16^. However, there is a lack of evidence regarding medication use during pregnancy and lactation, and the potential positive and negative effects it may have on pregnant women and their offspring. This situation directly impacts HCP clinical practice, given the challenges encountered when prescribing during these periods^17^, and taking into account that women rely principally on HCP advice when taking medication.

Therefore, the aim of this study is to identify the perceptions and needs related to the use of medication, vaccination, and alternative medicine during pregnancy and breastfeeding in Catalonia. Additionally, it seeks to understand where pregnant and breastfeeding women turn to and how they seek information when they have doubts regarding the use of medication, vaccination, and alternative medicine.

## METHODS

### Design

This is a descriptive cross-sectional study using data from a survey on opinions and feelings about the use of medicines during pregnancy and postpartum, including breastfeeding, with a gender perspective.

### Populations and recruitment

We non-randomly recruited 903 pregnant and recently postpartum women who attended the Sexual and Reproductive Health Care Services (*Atenció a la Salut Sexual i Reproductiva*, ASSIR) of Catalan Primary Care between November 2024 and June 2025. Inclusion criteria for participating in the survey were being aged 15–49 years, being pregnant or recently postpartum, and receiving pregnancy follow-up care at ASSIR. The inclusion criterion for this study was having available information on the gestational stage. We excluded 14 women who did not have information on the gestational stage. The final sample included 889 pregnant women who had recently given birth from the Catalan Primary Care Service.

### Instrument

The instrument used was an *ad hoc* questionnaire developed following the thematic framework identified in an earlier qualitative study conducted as part of the same research project, which is not yet published^18^. The questionnaire was developed for this study and evaluated by a panel of experts to ensure content validity and clarity, and a pilot test was conducted (**Supplementary file**). Nevertheless, a formal reliability assessment was not performed. It included 21 closed-ended questions, 4 closed-ended questions with an open ‘other’ option, 2 multiple-choice items with an open ‘other’ option, and 1 open-ended question addressing the use of medicines during pregnancy and breastfeeding, alternative medicine during pregnancy, and vaccination during pregnancy and breastfeeding. In addition, it collected sociodemographic characteristics, general health information, and pregnancy-related data. The questionnaire was available in Catalan and Spanish and was administered through the REDCap (Research Electronic Data Capture) platform by the participants themselves or via direct interview by the ASSIR nursing team. Study data were collected and managed using REDCap tools, hosted at Fundació Institut Universitari per a la recerca a l’Atenció Primària de Salut Jordi Gol i Gurina (IDIAPJGol)^19,20^. REDCap is a secure, web-based software platform designed to support data capture for research studies, providing: 1) an intuitive interface for validated data capture; 2) audit trails for tracking data manipulation and export procedures; 3) automated export procedures for seamless data downloads to common statistical packages; and 4) procedures for data integration and interoperability with external sources.

### Opinions and feelings about the use of medications during pregnancy

The study collected data on women’s opinions and feelings about the use of medicines during pregnancy through four questions addressing: how decisions to take medicines were made (doctor prescription or recommendation, advice from family or friends, or self-decision); sources consulted for information on medication risks (health professionals, the internet, medicine leaflets, advice from family or friends, or no consultation); whether conflicts arose between healthcare advice and personal research; and feelings about taking medicines during pregnancy (safe and confident, concerned but necessary, uncomfortable but necessary, uncomfortable and considered unnecessary, or not taking medicines).

### Opinions and feelings of complementary treatments during pregnancy

Data on opinions and experiences with alternative medicine and treatments during pregnancy were gathered through questions on whether vitamin supplements were used (doctor-prescribed, self-chosen, or not used) and on overall experiences with alternative medicine and treatments, ranging from very positive to very negative, including an option for non-use.

### Opinions and feelings about vaccination during pregnancy

Data on opinions and attitudes toward vaccination during pregnancy were collected using questions on the perceived need for pre-pregnancy vaccination consultations, agreement with vaccination during pregnancy, and willingness to receive specific types of vaccines, including those protecting the mother, the baby, both, or none.

### Opinions and feelings about medication use during breastfeeding

Data on opinions and practices regarding medication use during breastfeeding were collected through questions addressing: patterns of medication use for acute and chronic conditions; decision-making about taking medicines; sources consulted for information on medication risks; and whether conflicts were experienced between healthcare professional recommendations and personal research.

### Data on general health status, pregnancy, breastfeeding, and medication use during pregnancy

General health was self-rated on a six-point scale from excellent to very bad. Pregnancy-related data included the gestational window and the type of healthcare service providing prenatal care. Breastfeeding data captured whether women had breastfed or intended to do so. Medication use during pregnancy was assessed by identifying health conditions or symptoms requiring treatment, with predefined symptom categories and additional conditions derived from open-ended responses. Finally, the use of alternative medicine and therapies during pregnancy was recorded through yes/no questions covering various therapies, with further categories identified from open responses.

### Sociodemographic data

Sociodemographic data included age group, employment status, family structure, education level, and city population size. Age was categorized in five-year intervals and subsequently recoded as 15–24, 25–39, and 40–49 years for the present analysis. Employment status, family structure, and education level were collected with multiple response options and subsequently recoded into broader categories for analysis. City population size was classified as <10000 or ≥10000 inhabitants, while the ASSIR Center attended was recorded but not used in the analysis (Table 1).

**Table 1:** Sociodemographic, health, and pregnancy

*Characteristics*	*n (%)*
**Age** (years)	
15–24	34 (3.8)
25–39	772 (86.8)
40–49	83 (9.3)
**Employment status**	
Working	706 (80.3)
On medical leave or pensioner	78 (8.9)
Unemployment	77 (8.8)
Student	8 (0.9)
Not working	10 (1.1)
Missing	10
**Family structure**	
Couple	430 (49.1)
Couple with sons/daughters	407 (46.5)
Single mother	31 (3.5)
Family or community	8 (0.9)
Missing	13
**Education level**	
Primary education or lower	20 (2.3)
Secondary and upper education	83 (9.4)
Vocational training	215 (24.3)
University education	566 (64.0)
Missing	5
**City population**	
<10000	563 (64.4)
≥10000	311 (35.6)
Missing	15
**General health status**	
Excellent	123 (14.0)
Very good	435 (49.5)
Good	290 (33.0)
Regular	27 (3.1)
Bad/very bad	4 (0.5)
Missing	10
**Gestational window**	
First half	72 (8.1)
Second half	486 (54.7)
Postpartum period	331 (37.2)
**Healthcare service**	
Public	492 (55.3)
Hospital	114 (12.8)
Public/private	319 (35.9)
Private	61 (6.9)
**Received breastfeeding advice or thought about doing so?**	799 (92.4)
Missing	24

### Sample size

Sample size calculation was not performed because this was a descriptive study. Since the objective was to characterize the population rather than to test hypotheses or estimate associations, statistical power was not required. The study included all eligible participants recruited during the data collection period.

### Statistical analysis

We present the sociodemographic, pregnancy-related, breastfeeding, general health, and medication use characteristics during pregnancy as absolute frequencies and percentages. We also describe opinions and feelings about medication use during pregnancy, alternative medicine and treatments during pregnancy, and vaccination during pregnancy, as well as medication use during breastfeeding, all by absolute frequency and percentage. We describe the variables overall and stratified by age group, education level, and gestational window. In addition, we explore differences between groups using the chi-squared test or Fisher’s exact test, depending on whether the assumptions were met.

## RESULTS

A total of 903 pregnant and recently postpartum women were included in the study. Most participants were aged 30–34 years (40.2%), employed (79.4%), living with a partner (48.4%), and had a university education (63.7%). Over half (63.3%) lived in towns with <10000 inhabitants. Nearly half (48.9%) rated their general health as very good, 54.7% were in the second half of pregnancy, and 55.3% received pregnancy monitoring in the public healthcare service. The majority (89.9%) reported currently breastfeeding or intending to do so (Table 1).

**Figure 1:**
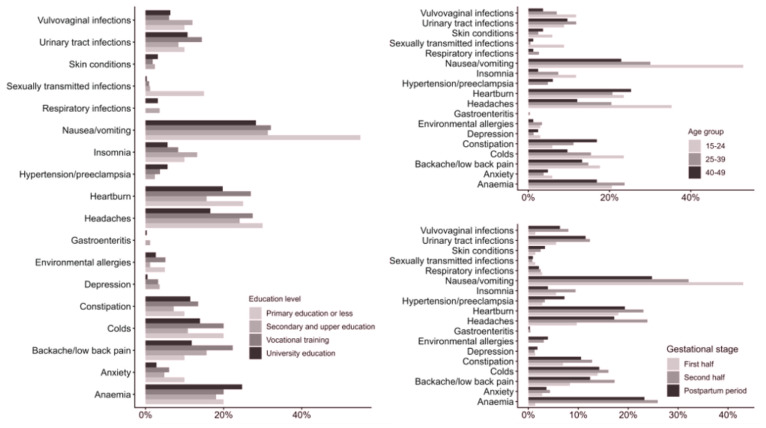
Percentages of health problems and symptoms during pregnancy stratified by education level, age group, and gestational stage

**Figure 2:**
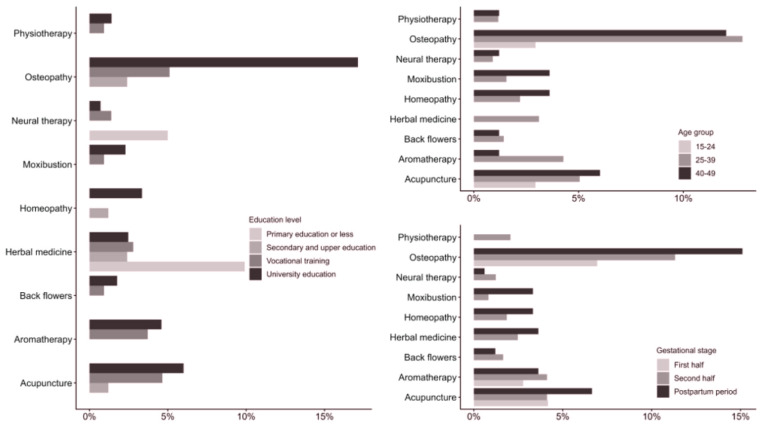
Percentages of use of alternative therapies stratified by education level, age group and gestational stage

Table 2 shows the health situation during pregnancy; a total of 59.3% of participants were diagnosed with a chronic illness, a health problem requiring closer monitoring, or minor symptoms that needed medication during pregnancy. In this sense, the most common symptoms and sensations requiring medication among pregnant women, affecting more than one in ten, were nausea and vomiting (30.3%), anemia (22.9%), heartburn (21.3%), headaches (20.2%), colds (15.1%), backache or low back pain (14.7%), constipation (11.5%), and urinary tract infections (11.5%). Women in the first half of pregnancy present more nausea (43.1%), in the second half of pregnancy, they present more anemia (25.9%), headache (23.9%), and insomnia (9.5%) (Figure 1 and Supplementary file Table 2). Table 2 shows that a total of 76.4% of participants have taken vitamin supplements prescribed by a physician. Table 2 also contains the information about the experience with alternative medicine, being more positive among the women with university studies (33.2%). Nonetheless, 66.6% of participants reported not using alternative medicine and therapies (Table 2). Osteopathy is the most used alternative medicine and therapy during pregnancy (12.4%), and participants with university studies were the ones who consumed the most alternative medicines (17.1%) (Figure 2 and Supplementary file Table 3).

**Table 2:** Health situation, vitamins use and experience of complementary therapies during pregnancy overall and stratified by age group, education level, and gestational stage

*All*	*Age group*					*Education level*						*Gestational window*				
	*A1*	*A2*	*A3*	*p*	*q^a^*	*E1*	*E2*	*E3*	*E4*	*p*	*q_a_*	*G1*	*G2*	*G3*	*p*	*q_a_*
**Total**, n																
889	34	772	83			20	83	215	566			72	486	331		
**Which of these three situations best fits your situation?**																
				0.155^b^	0.466					0.968^c^	0.968				0.259^c^	0.461
I had a chronic illness that required significant monitoring during pregnancy																
253 (28.6)	10 (29.4)	227 (29.6)	16 (19.3)			4 (20.0)	23 (27.7)	66 (31.0)	159 (28.2)			22 (30.6)	151 (31.2)	80 (24.3)		
I had a health problem that required medical visits during pregnancy																
201 (22.7)	9 (26.5)	169 (22.0)	23 (27.7)			4 (20.0)	18 (21.7)	47 (22.1)	131 (23.2)			12 (16.7)	106 (21.9)	83 (25.2)		
I had minor symptoms and took medication during pregnancy																
69 (7.8)	0 (0.0)	59 (7.7)	10 (12.0)			1 (5.0)	5 (6.0)	18 (8.5)	45 (8.0)			8 (11.1)	33 (6.8)	28 (8.5)		
I did not experience any of these things																
362 (40.9)	15 (44.1)	313 (40.8)	34 (41.0)			11 (55.0)	37 (44.6)	82 (38.5)	229 (40.6)			30 (41.7)	194 (40.1)	138 (41.9)		
Missing																
4	0	4	0			0	0	2	2			0	2	2		
**Have you taken vitamins supplements during pregnancy?**																
				0.608^b^	0.608					0.252^b^	0.378				0.461^b^	0.461
Prescribed by doctor																
679 (90.7)	26 (86.7)	581 (90.6)	72 (92.3)			18 (100.0)	66 (85.7)	169 (90.4)	422 (91.3)			52 (89.7)	381 (91.8)	246 (89.1)		
Own choice																
0 (0.0)	0 (0.0)	0 (0.0)	0 (0.0)			0 (0.0)	0 (0.0)	0 (0.0)	0 (0.0)			0 (0.0)	0 (0.0)	0 (0.0)		
Not taken any																
70 (9.3)	4 (13.3)	60 (9.4)	6 (7.7)			0 (0.0)	11 (14.3)	18 (9.6)	40 (8.7)			6 (10.3)	34 (8.2)	30 (10.9)		
Missing																
140	4	131	5			2	6	28	104			14	71	55		
**How has your experience been in use complementary treatments during pregnancy?**																
				0.581^c^	0.608					<0.001^c^	<0.001				0.325^c^	0.461
Very positive																
107 (13.0)	2 (6.5)	95 (13.3)	10 (13.2)			2 (13.3)	2 (2.5)	19 (9.4)	84 (16.2)			8 (12.5)	63 (13.9)	36 (11.9)		
Positive																
118 (14.4)	1 (3.2)	106 (14.9)	11 (14.5)			2 (13.3)	3 (3.8)	24 (11.8)	88 (17.0)			5 (7.8)	63 (13.9)	50 (16.5)		
Neutral																
46 (5.6)	2 (6.5)	41 (5.8)	3 (3.9)			1 (6.7)	2 (2.5)	10 (4.9)	32 (6.2)			5 (7.8)	25 (5.5)	16 (5.3)		
Negative/very negative																
3 (0.4)	0 (0.0)	3 (0.4)	0 (0.0)			0 (0.0)	0 (0.0)	1 (0.5)	2 (0.4)			0 (0.0)	0 (0.0)	3 (1.0)		
Did not use them																
546 (66.6)	26 (83.9)	468 (65.6)	52 (68.4)			10 (66.7)	72 (91.1)	149 (73.4)	313 (60.3)			46 (71.9)	302 (66.7)	198 (65.3)		
Missing																
69	3	59	7			5	4	12	47			8	33	28		

Data are presented as n (%). A1: 15–24, A2: 25–39, A3: 40–49 years. E1: primary or lower, E2: secondary and higher, E3: vocational training, E4: university. G1: first half, G2: second half, G3: postpartum.

a False discovery rate correction for multiple testing.

b Fisher’s exact test.

c Fisher’s exact test for count data with simulated p-value (based on 10000 replicates).

Table 3 presents the decision-making processes, information sources, and perceptions regarding the use of medicines during pregnancy. Overall, 84.6% of women reported that they decided to take medicines according to a prescription and/or recommendation from healthcare professionals. Additionally, 76.6% consulted healthcare professionals about the risks associated with medicine use during pregnancy, 72.0% reported no conflict between their own research and professional recommendations, and 51.7% felt safe and confident when taking medicines during pregnancy. The 40–49 years age group was the one that consulted more with HCPs regarding the medication they took (87.2%), and the younger group, aged 15–24 years, was the one that consulted more with the medicine leaflet (21.2%) (Table 3).

**Table 3:** Decision-making, information sources, and perceptions regarding the use of medicines during pregnancy overall and stratified by age group, education level and gestational stage

*All*	*Age group*					*Education level*						*Gestational window*				
	*A1*	*A2*	*A3*	*p*	*q^a^*	*E1*	*E2*	*E3*	*E4*	*p*	*q^a^*	*G1*	*G2*	*G3*	*p*	*q^a^*
**Total**, n																
889	34	772	83			20	83	215	566			72	486	331		
**If you took a medicine during pregnancy how did you make the decision?**																
				0.832^b^	0.832					0.587^b^	0.587				0.025^b^	0.050
Prescription and/or recommendation from healthcare professionals																
752 (94.6)	30 (93.8)	652 (94.5)	70 (95.9)			17 (94.4)	72 (93.5)	194 (96.5)	464 (93.9)			54 (88.5)	421 (95.9)	277 (93.9)		
Recommendation from pharmacists																
17 (2.1)	1 (3.1)	14 (2.0)	2 (2.7)			0 (0.0)	1 (1.3)	2 (1.0)	14 (2.8)			1 (1.6)	6 (1.4)	10 (3.4)		
Advice from family or friends																
3 (0.4)	0 (0.0)	3 (0.4)	0 (0.0)			0 (0.0)	0 (0.0)	0 (0.0)	3 (0.6)			0 (0.0)	1 (0.2)	2 (0.7)		
Own decision																
23 (2.9)	1 (3.1)	21 (3.0)	1 (1.4)			1 (5.6)	4 (5.2)	5 (2.5)	13 (2.6)			6 (9.8)	11 (2.5)	6 (2.0)		
Missing																
94	2	82	10			2	6	14	72			11	47	36		
**Where have you consulted about the risks of medicines?**																
				0.002^b^	0.006					0.108^d^	0.216				0.084^d^	0.084
Health professionals																
681 (80.3)	23 (69.7)	590 (80.1)	68 (87.2)			14 (77.8)	65 (80.2)	166 (79.0)	433 (81.1)			54 (79.4)	386 (83.7)	241 (75.5)		
The Internet																
69 (8.1)	1 (3.0)	62 (8.4)	6 (7.7)			0 (0.0)	5 (6.2)	16 (7.6)	48 (9.0)			5 (7.4)	35 (7.6)	29 (9.1)		
The medicine leaflet																
69 (8.1)	7 (21.2)	62 (8.4)	0 (0.0)			2 (11.1)	5 (6.2)	19 (9.0)	41 (7.7)			6 (8.8)	25 (5.4)	38 (11.9)		
Advice from family or friends																
4 (0.5)	1 (3.0)	2 (0.3)	1 (1.3)			1 (5.6)	0 (0.0)	1 (0.5)	2 (0.4)			0 (0.0)	2 (0.4)	2 (0.6)		
I did not consult about the risks																
25 (2.9)	1 (3.0)	21 (2.8)	3 (3.8)			1 (5.6)	6 (7.4)	8 (3.8)	10 (1.9)			3 (4.4)	13 (2.8)	9 (2.8)		
Missing																
41	1	35	5			2	2	5	32			4	25	12		
**I’ve experienced any conflict between my healthcare professional recommended and what I’ve researched on my own about the use of medicines during pregnancy**																
226 (26.1)	6 (19.4)	194 (25.7)	26 (32.1)	0.317^c^	0.423	3 (15.8)	12 (14.8)	49 (23.3)	161 (29.2)	0.017^b^	0.069	18 (25.7)	94 (19.9)	114 (35.3)	<0.001^c^	<0.001
Missing																
23	3	18	2			1	2	5	15			2	13	8		
**How did you feel about taking medicines during pregnancy?**																
				0.109^b^	0.218					0.565^d^	0.587				0.054^d^	0.071
Save and confident																
460 (55.5)	21 (65.6)	402 (55.8)	37 (48.7)			13 (72.2)	50 (64.9)	110 (53.1)	284 (54.4)			36 (53.7)	264 (58.8)	160 (51.1)		
Concerned but it was necessary																
218 (26.3)	8 (25.0)	189 (26.2)	21 (27.6)			5 (27.8)	19 (24.7)	60 (29.0)	132 (25.3)			16 (23.9)	118 (26.3)	84 (26.8)		
Uncomfortable but it was necessary																
106 (12.8)	2 (6.3)	96 (13.3)	8 (10.5)			0 (0.0)	5 (6.5)	25 (12.1)	76 (14.6)			7 (10.4)	51 (11.4)	48 (15.3)		
Uncomfortable and I believe that it was unnecessary																
11 (1.3)	0 (0.0)	7 (1.0)	4 (5.3)			0 (0.0)	1 (1.3)	3 (1.4)	7 (1.3)			1 (1.5)	5 (1.1)	5 (1.6)		
Did not take																
34 (4.1)	1 (3.1)	27 (3.7)	6 (7.9)			0 (0.0)	2 (2.6)	9 (4.3)	23 (4.4)			7 (10.4)	11 (2.4)	16 (5.1)		
Missing																
60	2	51	7			2	6	8	44			5	37	18		

Data are presented as n (%). A1: 15–24, A2: 25–39, A3: 40–49 years. E1: primary or lower, E2: secondary and higher, E3: vocational training, E4: university. G1: first half, G2: second half, G3: postpartum.

a False discovery rate correction for multiple testing.

b Fisher’s exact test.

c Pearson’s chi-squared test.

d Fisher’s exact test for count data with simulated p-value (based on 10000 replicates).

Table 4 presents attitudes and opinions regarding vaccination before and during pregnancy. Overall, 64.6% of participants considered it necessary to receive information from healthcare professionals about vaccination before becoming pregnant, 83.4% agreed with getting vaccinated during pregnancy, and 65.6% were willing to receive vaccines both to reduce the risk of disease during pregnancy and to prevent disease in the baby. During the first half of pregnancy, women present more reticence to get vaccinated (26.1%) (Table 4).

**Table 4:** Attitudes and opinions regarding vaccination before and during pregnancy overall and strataified by age group, eduation level and gestational stage

*All*	*Age group*					*Education level*						*Gestational window*				
	*A1*	*A2*	*A3*	*p*	*q^a^*	*E1*	*E2*	*E3*	*E4*	*p*	*q^a^*	*G1*	*G2*	*G3*	*p*	*q^a^*
**Total**, n																
889	34	772	83			20	83	215	566			72	486	331		
**Do you consider it necessary to have a medical consultation before becoming pregnant to ensure that you have had all the necessary vaccinations?**																
				0.427^b^	0.641					0.478d	0.478				0.646^d^	0.646
Yes																
574 (65.2)	27 (79.4)	496 (64.8)	51 (63.0)		16 (84.2)	55 (66.3)	136 (64.5)	364 (64.8)			43 (60.6)	310 (64.3)	221 (67.6)			
No																
133 (15.1)	2 (5.9)	119 (15.6)	12 (14.8)			1 (5.3)	11 (13.3)	27 (12.8)	93 (16.5)			10 (14.1)	76 (15.8)	47 (14.4)		
Don’t know																
173 (19.7)	5 (14.7)	150 (19.6)	18 (22.2)			2 (10.5)	17 (20.5)	48 (22.7)	105 (18.7)			18 (25.4)	96 (19.9)	59 (18.0)		
Missing																
9	0	7	2			1	0	4	4			1	4	4		
**I agree with getting vaccinated during pregnancy**																
741 (86.1)	28 (87.5)	645 (86.2)	68 (84.0)	0.824^c^	0.824	18 (94.7)	65 (82.3)	176 (84.2)	478 (87.1)	0.392c	0.478	51 (73.9)	417 (88.3)	273 (85.3)	0.005^b^	0.007
Missing																
28	2	24	2			1	4	6	17			3	14	11		
**Which vaccinations would you be willing to have?**																
				0.142^c^	0.427					<0.001d	<0.001				<0.001^d^	<0.001
Those necessary to reduce the risk of disease during pregnancy																
90 (10.3)	5 (15.2)	78 (10.3)	7 (8.4)			3 (15.0)	13 (15.9)	36 (16.9)	37 (6.7)			16 (22.5)	52 (10.9)	22 (6.7)		
Those that prevent disease in the baby																
172 (19.6)	5 (15.2)	157 (20.7)	10 (12.0)			4 (20.0)	16 (19.5)	35 (16.4)	115 (20.7)			5 (7.0)	92 (19.2)	75 (23.0)		
Both of the above																
583 (66.6)	20 (60.6)	501 (65.9)	62 (74.7)			13 (65.0)	45 (54.9)	132 (62.0)	391 (70.3)			43 (60.6)	318 (66.4)	222 (68.1)		
None																
31 (3.5)	3 (9.1)	24 (3.2)	4 (4.8)			0 (0.0)	8 (9.8)	10 (4.7)	13 (2.3)			7 (9.9)	17 (3.5)	7 (2.1)		
Missing																
13	1	12	0			0	1	2	10			1	7	5		

Data are presented as n (%). A1: 15–24, A2: 25–39, A3: 40–49 years. E1: primary or lower, E2: secondary and higher, E3: vocational training, E4: university. G1: first half, G2: second half, G3: postpartum.

a False discovery rate correction for multiple testing.

b Fisher’s exact test.

c Pearson’s chi-squared test.

d Fisher’s exact test for count data with simulated p-value (based on 10000 replicates).

Table 5 shows the decision-making processes, information sources, and perceptions regarding the use of medicines during breastfeeding, with a 40.3% of participants who took medication, doing so according to the usual schedule or when it was due. Moreover, 48.8% used medicines only if necessary and prescribed by healthcare professionals. A total of 81.4% made decisions about medicine use based on prescriptions or healthcare recommendations. Additionally, 66.1% sought information on the risks of medication use during breastfeeding from healthcare professionals. Finally, 70.5% did not experience conflicts between the information they sought and the recommendations they received. Education level affected the way women related to their HCP when making decisions during pregnancy; those with a higher level of education (university) showed more reticence and conflict with their HCP (25.1%) (Table 5).

**Table 5:** Medication use, decision-making, and information-seeking behavior during breastfeeding overall and stratified by age group, education level and gestational stage

*All*	*Age group*					*Education level*						*Gestational window*				
	*A1*	*A2*	*A3*	*p*	*q^a^*	*E1*	*E2*	*E3*	*E4*	*p*	*q^a^*	*G1*	*G2*	*G3*	*p*	*q^a^*
**Total**, n																
889	34	772	83			20	83	215	566			72	486	331		
**If you took any medication while breastfeeding due to health problem, which situation best fits your case?**																
				0.060^b^	0.173					0.672^d^	0.676				<0.001^d^	<0.001
I take my medication according to the usual schedule or when it is due																
358 (42.7)	12 (36.4)	306 (42.0)	40 (51.9)			9 (47.4)	31 (39.7)	83 (40.9)	233 (43.6)			23 (32.9)	164 (36.4)	171 (53.8)		
If I need take medication I separate it from breastfeeding																
117 (13.9)	6 (18.2)	108 (14.8)	3 (3.9)			3 (15.8)	10 (12.8)	30 (14.8)	74 (13.9)			12 (17.1)	79 (17.5)	26 (8.2)		
I avoid taking any medication																
292 (34.8)	10 (30.3)	254 (34.8)	28 (36.4)			4 (21.1)	28 (35.9)	69 (34.0)	189 (35.4)			28 (40.0)	164 (36.4)	100 (31.4)		
I avoid taking any medication, and I do not take medication																
72 (8.6)	5 (15.2)	61 (8.4)	6 (7.8)			3 (15.8)	9 (11.5)	21 (10.3)	38 (7.1)			7 (10.0)	44 (9.8)	21 (6.6)		
Missing																
50	1	43	6			1	5	12	32			2	35	13		
**If I took chronic medication during breastfeeding, which situation best fits your case?**																
				0.152^b^	0.190					0.001^d^	0.003				0.572^d^	0.572
I have no problem taking medicines during breastfeeding																
56 (6.6)	6 (17.6)	42 (5.7)	8 (10.1)			5 (25.0)	7 (9.0)	15 (7.4)	29 (5.4)			7 (10.1)	27 (5.9)	22 (6.8)		
I take medication if it is necessary and it has been prescribed by health professional																
434 (51.4)	15 (44.1)	383 (52.3)	36 (45.6)			6 (30.0)	37 (47.4)	126 (61.8)	264 (49.1)			35 (50.7)	235 (51.8)	164 (50.9)		
I avoid taking any medication while I breastfeeding																
80 (9.5)	3 (8.8)	68 (9.3)	9 (11.4)			2 (10.0)	8 (10.3)	19 (9.3)	50 (9.3)			6 (8.7)	37 (8.1)	37 (11.5)		
I don’t take chronic medication																
275 (32.5)	10 (29.4)	239 (32.7)	26 (32.9)			7 (35.0)	26 (33.3)	44 (21.6)	195 (36.2)			21 (30.4)	155 (34.1)	99 (30.7)		
Missing																
44	0	40	4			0	5	11	28			3	32	9		
**If you took a medication during breastfeeding how you took the decision?**																
				0.864^b^	0.864					0.676^b^	0.676				0.029^b^	0.036
Prescription and/or recommendation from healthcare professionals																
724 (95.6)	28 (96.6)	627 (95.4)	69 (97.2)			18 (94.7)	65 (95.6)	182 (97.8)	456 (95.0)			55 (93.2)	377 (97.4)	292 (93.9)		
Recommendation from pharmacists																
9 (1.2)	0 (0.0)	8 (1.2)	1 (1.4)			0 (0.0)	0 (0.0)	1 (0.5)	8 (1.7)			1 (1.7)	1 (0.3)	7 (2.3)		
Advice from family or friends																
2 (0.3)	0 (0.0)	2 (0.3)	0 (0.0)			0 (0.0)	0 (0.0)	0 (0.0)	2 (0.4)			0 (0.0)	2 (0.5)	0 (0.0)		
Own decision																
22 (2.9)	1 (3.4)	20 (3.0)	1 (1.4)			1 (5.3)	3 (4.4)	3 (1.6)	14 (2.9)			3 (5.1)	7 (1.8)	12 (3.9)		
Missing																
132	5	115	12			1	15	29	86			13	99	20		
**How do you query about the risks of taking medicines during breastfeeding?**																
				0.104^b^	0.173					<0.001^b^	<0.00^1^				<0.001^b^	<0.001
I queried it to health professionals																
588 (73.7)	21 (67.7)	507 (73.1)	60 (82.2)			15 (78.9)	50 (67.6)	146 (75.6)	375 (73.8)			49 (76.6)	331 (79.2)	208 (65.8)		
I searched it in internet																
41 (5.1)	1 (3.2)	36 (5.2)	4 (5.5)			0 (0.0)	2 (2.7)	11 (5.7)	28 (5.5)			3 (4.7)	20 (4.8)	18 (5.7)		
I reviewed the leaflet																
127 (15.9)	4 (12.9)	117 (16.9)	6 (8.2)			2 (10.5)	6 (8.1)	27 (14.0)	91 (17.9)			7 (10.9)	37 (8.9)	83 (26.3)		
I did not query																
42 (5.3)	5 (16.1)	34 (4.9)	3 (4.1)			2 (10.5)	16 (21.6)	9 (4.7)	14 (2.8)			5 (7.8)	30 (7.2)	7 (2.2)		
Missing																
91	3	78	10			1	9	22	58			8	68	15		
**I’ve ever experienced conflicts between what my healthcare professional recommended and what I’ve researched on my own about the use of medicines during breastfeeding**																
182 (22.5)	4 (12.9)	155 (22.0)	23 (31.1)	0.088^c^	0.173	2 (10.5)	9 (11.8)	39 (20.2)	130 (25.1)	0.026^b^	0.043	15 (22.7)	67 (15.8)	100 (31.3)	<0.001^c^	<0.001
Missing																
80	3	68	9			1	7	22	49			6	62	12		

Data are presented as n (%). A1: 15–24, A2: 25–39, A3: 40–49 years. E1: primary or lower, E2: secondary and higher, E3: vocational training, E4: university. G1: first half, G2: second half, G3: postpartum.

a False discovery rate correction for multiple testing.

b Fisher’s exact test.

c Pearson’s chi-squared test.

d Fisher’s exact test for count data with simulated p-value (based on 10000 replicates).

## DISCUSSION

A total of 903 pregnant and recently postpartum women were included. More than half of them experienced a chronic illness, a health condition requiring closer monitoring, or minor symptoms that required medication during pregnancy. Most women reported that they decided to take medicines based on a prescription or recommendation from healthcare professionals. About half of the women felt safe and confident when taking medicines during pregnancy. Nearly two-thirds considered it necessary to receive information from healthcare professionals about vaccination before becoming pregnant. Among the participants who took medication, around four in ten did so according to the usual schedule or when it was due. Almost half used medicines only when necessary and as prescribed by HCP.

As evidenced by this study and previous literature, women show caution when taking medicines during pregnancy and breastfeeding^4,21^. Even so, in many cases, medication is necessary to ensure the mother’s good health and/or the proper development of the baby^22^. In this study, 59.3% of the participants had some type of chronic or acute condition that, in many cases, requires medication, which makes it imperative to improve the existing evidence on the use of medicines during pregnancy and breastfeeding^17^. During breastfeeding, most participants also tended to avoid medication, and when necessary, they only took those recommended by healthcare professionals.

Vaccination was seen as necessary during pregnancy by 83.4% of the study participants, who considered it beneficial both for reducing health problems in the mother during pregnancy and for the baby’s well-being, being equally high in all ages and in education levels. In the Catalan context, the vaccination recommendations of the Department of Health include the pertussis vaccine, the flu vaccine, and the COVID-19 vaccine^23^. Even so, previous evidence has shown that fears exist regarding the COVID-19 vaccine due to the limited prior research and the uncertainty surrounding its possible effects now and in the future^24,25^. Regarding vaccination, there is a very clear protocol in Catalonia, on which vaccines are recommended and which are not during pregnancy, something that may influence the perceived safety of using them during pregnancy^23^. It is also worth considering that the limited information on vaccination and medicines during pregnancy and breastfeeding can be explained due to ethical and legislative considerations and limitations to conduct pharmacological research with pregnant and breastfeeding women^26^, but also due to the prevailing androcentric perspective on health research^27^. Therefore, it is imperative to advance in generating evidence on the safety of medicines and vaccines.

It is also increasingly common to use different products and therapies from alternative medicine and treatments (e.g. acupuncture, homeopathy, osteopathy, aromatherapy, etc.), especially among women^28-30^. This interest in using products considered more natural is part of a series of lifestyle changes that lead to viewing these products as less invasive options for health issues^28^. Despite this perception, some alternative medicines may have teratogenic effects and/or interact with other medications, making it important to increase the existing evidence on them and take them into account during pregnancy in order to recommend those that may be beneficial and avoid those that are dangerous^28,31^. Our study shows that 38.6% of the participants had consumed or used some alternative medicine and therapies, with those with university studies being the ones who consumed more of these products and therapies. Therefore, the use of herbal products in our study is very low, and most participants had undergone osteopathy or acupuncture, two techniques that are safe because they do not involve taking any product. However, it would be interesting to strengthen the existing evidence on the benefits of these two techniques and to incorporate them into the healthcare system’s service, so that all women can access them regardless of their socioeconomic status.

This interest in the pursuit of products perceived as more natural is also consistent with the fact that, historically, reproductive processes have at times been subject to unnecessary medicalization^6^. Consequently, many women seek to avoid the use of pharmaceutical treatments and may develop certain fears and reservations toward the recommendations of healthcare professionals. In this regard, it is relevant to address and further examine the processes of decision-making during pregnancy and breastfeeding. Most participants trusted the recommendations of healthcare professionals, which is consistent with previous evidence^33,34^. Some of them, in addition, also consulted the internet or the medication leaflet^35^. Although 25.4% of participants during pregnancy and 20.5% during breastfeeding experienced some conflict between their opinion and the recommendation of their health care professional regarding the use of medicines. Increasingly, more people seek information on their own during pregnancy and breastfeeding, which may partly contribute to these conflicts with healthcare professionals, especially considering that the majority of participants in this study had a university-level education^36^. In this sense, it is useful to work on shared decision-making to continue building the necessary trust to ensure good adherence and proper use of medication when needed, and to avoid misuse^37^.

### Strengths and limitations

This study has several limitations. First data were collected using an *ad hoc* questionnaire that, although carefully designed, was not previously validated, which may affect the reliability and comparability of the findings. Second, responses were self-reported, introducing the potential for recall and social desirability biases. Third, the sample was recruited non-randomly from participants attending ASSIR, which may limit the generalizability of the results to the wider population. In this line, most participants had university-level education, which may represent a limitation, as the sample may not be representative of the Catalan population. However, according to data from IDESCAT^38^, the majority of pregnant women in Catalonia have higher education, which may help explain the characteristics of our sample.

## CONCLUSIONS

This study highlights that a substantial proportion of pregnant, postpartum, and breastfeeding women experience health conditions that require pharmacological treatment during pregnancy and breastfeeding. Despite this, most participants demonstrated a cautious approach to medication use, prioritizing prescriptions and recommendations from HCP and avoiding unnecessary treatments. Trust in healthcare professionals emerged as a key factor guiding decision-making, both during pregnancy and breastfeeding, contributing to a general sense of safety and confidence when medication was required. The findings also indicate limited use of alternative therapies, with osteopathy being the most commonly used option, particularly among women with university degrees. Vaccination during pregnancy was widely accepted among participants, who recognized its benefits for both maternal and infant health. Overall, these results emphasize the central role of healthcare professionals in providing reliable information and working on shared decision-making. Strengthening communication and addressing potential conflicts between personal beliefs and professional recommendations may further improve medication adherence, appropriate use, and health outcomes for both mothers and their babies during pregnancy and breastfeeding.

## Supplementary Material



## Data Availability

The datasets generated and analyzed during the current study are available from the corresponding author on reasonable request.
