# Supplementary material for: Perceptions on the use of medicines, vaccines, and alternative medicine during pregnancy and breastfeeding: Population-based survey in Catalonia, Spain
Source: Eur J Midwifery. 2026 Jul 24;10:10.18332/ejm/225498. doi: 10.18332/ejm/225498 (PMC13401239; doi:10.18332/ejm/225498)
Supplement: Supplementary file 1 [file EJM-10-32-s1.pdf]

EUROPEAN JOURNAL
OF MIDWIFERY

**Supplementary file**

© 2026 Munné-Barellas B. et al.

**DOI:**
10.18332/ejm/225498

The content has been provided by the author(s) and has not been reviewed, verified, or endorsed by European Publishing. It may not have undergone peer review. The views, opinions, and recommendations expressed are solely those of the author(s) and do not necessarily reflect the position of European Publishing. European Publishing accepts no responsibility or liability for any consequences arising from the use of, or reliance on, this content.

**SURVEY – Medicines, complementary medicine and vaccination during pregnancy and breastfeeding**

You are pregnant or have recently had a baby. We would therefore like to ask you to complete this survey to find out how your Health has been during pregnancy:

Multiple choice

***Personal data***

We start the survey with some questions to find out its context

**1. How old is it?**

1. 15-19
2. 20-24
3. 25-29
4. 30-34
5. 35-39
6. 40-44
7. 45-49

**2. Which ASSIR have you ______________________________________**

**3. What is your occupational situation outside the maternity process?**

1. I work full time
2. I work part-time
1. Medical leave
2. Unemployment
3. I'm studying
1. Others: ________________

**4. Family situation**

1. Single-mother family
2. I live only with a partner
3. I live with my partner plus 1 child
4. I live with the couple plus 2 children
5. I live with my partner and 3 or more children
6. Others: ______________________

**5. What studies does he have?**

1. Primary education
2. Secondary education
3. Vocational training (intermediate/higher level)
1. University studies (bachelor's/bachelor's degree)
2. Postgraduate studies (master's/doctorate)
3. No studies completed
4. Others: ____________________

**6. Area of residence**

1. Large city (+10,000 inhabitants)
2. Small town/village (-10,000 inhabitants)

**7. How would you define your overall health:**

1. Excellent
2. Very good
3. Good
4. Regular
5. Bad

6. Very bad

*Pregnancy data*

***The following questions are related to your pregnancy process and medication use***

**1. Is this your first pregnancy?**

- Yes
- No

**2. At what stage of pregnancy/breastfeeding are you?**

- First half of pregnancy
- Second half pregnancy
- Puerperium (I've already had the baby)

**3. How did you monitor your pregnancy?**

- Audience: ASSIR
- Audience: Hospital (if at risk)
- Public-private
- Private

**4. Which of the 3 situations do you think best suits your situation?**

- I have a chronic illness that has required greater control throughout my pregnancy.
- I have had a health problem during pregnancy that has required visits.
- I have presented irrelevant symptoms and therefore I have not had to take medication during pregnancy. (if you check this one there is no longer the option to follow this blog)

**Needs around drugs**

**5. What symptoms/sensations have generated the need for medication?**

- Nausea and/or vomiting
- Heartburn
- Headache
- Insomnia
- Anxiety
- Depression
- Urinary tract infections
- Vulvovaginal infections
- Sexually transmitted infections
- Respiratory infections
- Muscle pain
- Colds
- Constipation
- Gastroenteritis
- Back pain/low back pain
- Environmental allergies
- Skin disorders
- Anaemia
- Others: ______________________________
- I have not had any health problems that have required medication

**6. If you had to take a drug during pregnancy, how did you make the decision?**

- Prescription and/or recommendation of a health professional
- Recommendation from the pharmacy or others
- Advice from family or friends

- By own decision

7. **Where can you find out about the risks of medicines?**
   - I have consulted health professionals
   - I searched the internet
   - I have reviewed the leaflet
   - Advice from family or friends
   - I have not consulted the risks

8. **Have you ever experienced a conflict between what your healthcare professionals recommend to you and what you have researched on your own about medications during pregnancy?**
   - Yes
   - No

9. **Have you taken vitamin supplements during pregnancy?**
   - Prescribed by the doctor
   - On your own initiative
   - I didn't take it

10. **How did you feel about taking medication during pregnancy?**
    - Safe and confident
    - Worried, but it was necessary
    - Uncomfortable, but it was necessary
    - Uncomfortable and I think it was not necessary
    - Others: ________________________________________

***Medicine/complementary treatments***
***Below we will ask you about the use of complementary medication during pregnancy***

11. **Have you used any complementary therapy during pregnancy?**
    - Acupuncture
    - Homeopathy
    - Aromatherapy
    - Osteopathy
    - Neural therapy
    - Moxibustion
    - Bach Flowers
    - Phytotherapy (medicinal herbs)
    - Others: ____________________________________________

12. **How has your experience of using natural treatments during pregnancy been?**
    - Very positive
    - Positive
    - Neutral
    - Negative
    - Very negative
    - I didn't take it

***Vaccination***
***The following questions deal with the use you have made of vaccination during pregnancy***

13. **Do you consider it necessary to have a prior visit before pregnancy to ensure that you have the necessary vaccinations?**
    - Yes

- No
- I don't know

**14. Are you okay with receiving vaccines during pregnancy?**

- Yes
- No

**15. What vaccines would you agree to get?**

- Necessary to reduce the risk of diseases during pregnancy
- To prevent diseases in the baby
- The two previous options
- None

***Breastfeeding***
***Below we ask you about breastfeeding and the use of medications during this period***

**16. Have you done or plan to breastfeed?**

- Yes
- No

**17. If you have to take any medication while breastfeeding due to a health problem, which option do you prefer?**

- I take the medication according to the normal schedule (or when it is appropriate)
- If I need to take a medication, I separate it from breastfeeding
- I avoid taking any medication
- I don't take medication, but I do take natural remedies

**18. If I have to take chronic medication during breastfeeding, which option do you think is more appropriate?**

- I have no problems taking medication while breastfeeding
    - I take medication if necessary and if it has been prescribed to me by a health professional
    - During breastfeeding I avoid taking any medication
    - I don't take chronic medication

**19. If you had to take a drug during breastfeeding, how did you make the decision?**

- Prescription and/or recommendation of a health professional
    - Pharmacy recommendation
    - Recommendation of people in the environment, family or friends
    - By own decision

**20. How do you inquire about the risks of medications during breastfeeding?**

- I have consulted health professionals
- I searched the internet
- I have reviewed the leaflet
- I have not consulted them

**21. Have you ever experienced a conflict between what your healthcare professional recommends and what you have researched on your own about breastfeeding medications?**

- Yes
- No

*Supplementary table 1: Health problems and symptoms during pregnancy overall and stratified age group, education level and gestational stage*

| | Overall | Age group | | | | | Educational level | | | | | | Pregnancy stage | | | | |
|---|---|---|---|---|---|---|---|---|---|---|---|---|---|---|---|---|---|
| **Characteristic** | **N = 889**[1] | **15-24** N = 34[1] | **25-39** N = 772[1] | **40-49** N = 83[1] | **p-value** | **q-value**[2] | **Primary education or less** N = 20[1] | **Secondary and upper education** N = 83[1] | **Vocational training** N = 215[1] | **University education** N = 566[1] | **p-value** | **q-value**[2] | **First half** N = 72[1] | **Second half** N = 486[1] | **Postpartum period** N = 331[1] | **p-value** | **q-value**[2] |
| Nausea/vomiting | 269 (30.3%) | 18 (52.9%) | 232 (30.1%) | 19 (22.9%) | 0.005[3] | 0.049 | 11 (55.0%) | 26 (31.3%) | 69 (32.1%) | 160 (28.3%) | 0.064[3] | 0.144 | 31 (43.1%) | 156 (32.1%) | 82 (24.8%) | 0.004[3] | 0.028 |
| Anaemia | 204 (22.9%) | 7 (20.6%) | 183 (23.7%) | 14 (16.9%) | 0.351[3] | 0.632 | 4 (20.0%) | 15 (18.1%) | 43 (20.0%) | 140 (24.7%) | 0.371[4] | 0.514 | 1 (1.4%) | 126 (25.9%) | 77 (23.3%) | <0.001[3] | <0.001 |
| Heartburn | 189 (21.3%) | 8 (23.5%) | 160 (20.7%) | 21 (25.3%) | 0.593[3] | 0.821 | 5 (25.0%) | 13 (15.7%) | 58 (27.0%) | 112 (19.8%) | 0.086[4] | 0.172 | 13 (18.1%) | 112 (23.0%) | 64 (19.3%) | 0.350[3] | 0.573 |
| Headaches | 180 (20.2%) | 12 (35.3%) | 158 (20.5%) | 10 (12.0%) | 0.016[3] | 0.097 | 6 (30.0%) | 20 (24.1%) | 59 (27.4%) | 94 (16.6%) | 0.003[4] | 0.021 | 7 (9.7%) | 116 (23.9%) | 57 (17.2%) | 0.005[3] | 0.028 |
| Backache/low back pain | 131 (14.7%) | 6 (17.6%) | 114 (14.8%) | 11 (13.3%) | 0.829[3] | 0.926 | 2 (10.0%) | 13 (15.7%) | 48 (22.3%) | 67 (11.8%) | 0.004[4] | 0.021 | 6 (8.3%) | 84 (17.3%) | 41 (12.4%) | 0.043[3] | 0.128 |
| Colds | 135 (15.2%) | 8 (23.5%) | 119 (15.4%) | 8 (9.6%) | 0.146[3] | 0.525 | 4 (20.0%) | 9 (10.8%) | 43 (20.0%) | 79 (14.0%) | 0.103[4] | 0.185 | 10 (13.9%) | 78 (16.0%) | 47 (14.2%) | 0.731[3] | 0.881 |
| Constipation | 102 (11.5%) | 2 (5.9%) | 86 (11.1%) | 14 (16.9%) | 0.189[4] | 0.530 | 2 (10.0%) | 6 (7.2%) | 29 (13.5%) | 65 (11.5%) | 0.522[4] | 0.553 | 5 (6.9%) | 62 (12.8%) | 35 (10.6%) | 0.286[3] | 0.514 |
| Urinary tract infections | 102 (11.5%) | 3 (8.8%) | 91 (11.8%) | 8 (9.6%) | 0.853[4] | 0.926 | 2 (10.0%) | 7 (8.4%) | 31 (14.4%) | 61 (10.8%) | 0.434[4] | 0.525 | 4 (5.6%) | 60 (12.3%) | 38 (11.5%) | 0.241[3] | 0.502 |

| | Overall | Age group | | | | | Educational level | | | | | | Pregnancy stage | | | | |
|---|---|---|---|---|---|---|---|---|---|---|---|---|---|---|---|---|---|
| **Characteristic** | **N = 889**[1] | **15-24** N = 34[1] | **25-39** N = 772[1] | **40-49** N = 83[1] | **p-value** | **q-value**[2] | **Primary education or less** N = 20[1] | **Secondary and upper education** N = 83[1] | **Vocational training** N = 215[1] | **University education** N = 566[1] | **p-value** | **q-value**[2] | **First half** N = 72[1] | **Second half** N = 486[1] | **Postpartum period** N = 331[1] | **p-value** | **q-value**[2] |
| Insomnia | 63 (7.1%) | 4 (11.8%) | 57 (7.4%) | 2 (2.4%) | 0.099[4] | 0.446 | 2 (10.0%) | 11 (13.3%) | 18 (8.4%) | 32 (5.7%) | 0.050[4] | 0.134 | 4 (5.6%) | 46 (9.5%) | 13 (3.9%) | 0.009[3] | 0.040 |
| Vulvovaginal infections | 61 (6.9%) | 4 (11.8%) | 54 (7.0%) | 3 (3.6%) | 0.265[4] | 0.530 | 2 (10.0%) | 10 (12.0%) | 13 (6.0%) | 36 (6.4%) | 0.200[4] | 0.300 | 1 (1.4%) | 39 (8.0%) | 21 (6.3%) | 0.094[4] | 0.241 |
| Hypertension/preeclampsia | 42 (4.7%) | 0 (0.0%) | 37 (4.8%) | 5 (6.0%) | 0.452[4] | 0.740 | 0 (0.0%) | 2 (2.4%) | 8 (3.7%) | 32 (5.7%) | 0.466[4] | 0.525 | 2 (2.8%) | 16 (3.3%) | 24 (7.3%) | 0.028[4] | 0.101 |
| Anxiety | 35 (3.9%) | 2 (5.9%) | 29 (3.8%) | 4 (4.8%) | 0.506[4] | 0.759 | 2 (10.0%) | 4 (4.8%) | 13 (6.0%) | 16 (2.8%) | 0.052[4] | 0.134 | 2 (2.8%) | 21 (4.3%) | 12 (3.6%) | 0.832[4] | 0.881 |
| Environmental allergies | 28 (3.1%) | 1 (2.9%) | 26 (3.4%) | 1 (1.2%) | 0.651[4] | 0.837 | 1 (5.0%) | 1 (1.2%) | 11 (5.1%) | 15 (2.7%) | 0.169[4] | 0.277 | 0 (0.0%) | 15 (3.1%) | 13 (3.9%) | 0.251[4] | 0.502 |
| Skin conditions | 24 (2.7%) | 2 (5.9%) | 19 (2.5%) | 3 (3.6%) | 0.260[4] | 0.530 | 0 (0.0%) | 2 (2.4%) | 4 (1.9%) | 18 (3.2%) | 0.800[4] | 0.800 | 1 (1.4%) | 12 (2.5%) | 11 (3.3%) | 0.690[4] | 0.881 |
| Respiratory infections | 21 (2.4%) | 0 (0.0%) | 20 (2.6%) | 1 (1.2%) | 0.874[4] | 0.926 | 0 (0.0%) | 3 (3.6%) | 0 (0.0%) | 18 (3.2%) | 0.018[4] | 0.066 | 2 (2.8%) | 12 (2.5%) | 7 (2.1%) | 0.788[4] | 0.881 |
| Depression | 13 (1.5%) | 1 (2.9%) | 10 (1.3%) | 2 (2.4%) | 0.263[4] | 0.530 | 0 (0.0%) | 3 (3.6%) | 7 (3.3%) | 3 (0.5%) | 0.010[4] | 0.044 | 1 (1.4%) | 6 (1.2%) | 6 (1.8%) | 0.757[4] | 0.881 |
| Sexually transmitted infections | 8 (0.9%) | 3 (8.8%) | 4 (0.5%) | 1 (1.2%) | 0.002[4] | 0.042 | 3 (15.0%) | 1 (1.2%) | 2 (0.9%) | 2 (0.4%) | <0.001[4] | 0.008 | 1 (1.4%) | 4 (0.8%) | 3 (0.9%) | 0.738[4] | 0.881 |
| Gastroenteritis | 3 (0.3%) | 0 (0.0%) | 3 (0.4%) | 0 (0.0%) | >0.999[4] | >0.999 | 0 (0.0%) | 1 (1.2%) | 0 (0.0%) | 2 (0.4%) | 0.438[4] | 0.525 | 0 (0.0%) | 2 (0.4%) | 1 (0.3%) | >0.999[4] | >0.999 |

[1]n (%)

[2]False discovery rate correction for multiple testing

[3]Pearson's Chi-squared test

| | Overall | Age group | | | | | Educational level | | | | | | Pregnancy stage | | | | |
|---|---|---|---|---|---|---|---|---|---|---|---|---|---|---|---|---|---|
| **Characteristic** | **N = 889**[1] | **15-24** N = 34[1] | **25-39** N = 772[1] | **40-49** N = 83[1] | **p-value** | **q-value**[2] | **Primary education or less** N = 20[1] | **Secondary and upper education** N = 83[1] | **Vocational training** N = 215[1] | **University education** N = 566[1] | **p-value** | **q-value**[2] | **First half** N = 72[1] | **Second half** N = 486[1] | **Postpartum period** N = 331[1] | **p-value** | **q-value**[2] |

[4]Fisher's exact test

Supplementary table 2: Use of alternative therapies during pregnancy overall and stratified by age group, education level and gestational stage

| | **Overall** | **Age group** | | | | | **Educational level** | | | | | | **Pregnancy stage** | | | | |
|---|---|---|---|---|---|---|---|---|---|---|---|---|---|---|---|---|---|
| **Characteristic** | **N = 889**[1] | **15-24** N = 34[1] | **25-39** N = 772[1] | **40-49** N = 83[1] | **p-value** | **q-value**[2] | **Primary education or less** N = 20[1] | **Secondary and upper education** N = 83[1] | **Vocational training** N = 215[1] | **University education** N = 566[1] | **p-value** | **q-value**[2] | **First half** N = 72[1] | **Second half** N = 486[1] | **Postpartum period** N = 331[1] | **p-value** | **q-value**[2] |
| Acupuncture | 45 (5.1%) | 1 (2.9%) | 39 (5.1%) | 5 (6.0%) | 0.819[3] | >0.999 | 0 (0.0%) | 1 (1.2%) | 10 (4.7%) | 34 (6.0%) | 0.250[3] | 0.375 | 3 (4.2%) | 20 (4.1%) | 22 (6.6%) | 0.262[3] | 0.393 |
| Homeopathy | 20 (2.2%) | 0 (0.0%) | 17 (2.2%) | 3 (3.6%) | 0.527[3] | 0.949 | 0 (0.0%) | 1 (1.2%) | 0 (0.0%) | 19 (3.4%) | 0.017[3] | 0.075 | 0 (0.0%) | 9 (1.9%) | 11 (3.3%) | 0.190[3] | 0.393 |
| Aromatherapy | 34 (3.8%) | 0 (0.0%) | 33 (4.3%) | 1 (1.2%) | 0.298[3] | 0.671 | 0 (0.0%) | 0 (0.0%) | 8 (3.7%) | 26 (4.6%) | 0.174[3] | 0.375 | 2 (2.8%) | 20 (4.1%) | 12 (3.6%) | 0.964[3] | 0.964 |
| Osteopathy | 110 (12.4%) | 1 (2.9%) | 99 (12.8%) | 10 (12.0%) | 0.239[3] | 0.671 | 0 (0.0%) | 2 (2.4%) | 11 (5.1%) | 97 (17.1%) | <0.001[3] | <0.001 | 5 (6.9%) | 55 (11.3%) | 50 (15.1%) | 0.094[4] | 0.281 |
| Neural therapy | 8 (0.9%) | 0 (0.0%) | 7 (0.9%) | 1 (1.2%) | 0.678[3] | >0.999 | 1 (5.0%) | 0 (0.0%) | 3 (1.4%) | 4 (0.7%) | 0.140[3] | 0.375 | 0 (0.0%) | 6 (1.2%) | 2 (0.6%) | 0.633[3] | 0.813 |
| Moxibustion | 15 (1.7%) | 0 (0.0%) | 12 (1.6%) | 3 (3.6%) | 0.254[3] | 0.671 | 0 (0.0%) | 0 (0.0%) | 2 (0.9%) | 13 (2.3%) | 0.438[3] | 0.564 | 0 (0.0%) | 4 (0.8%) | 11 (3.3%) | 0.020[3] | 0.092 |
| Back flowers | 12 (1.3%) | 0 (0.0%) | 11 (1.4%) | 1 (1.2%) | >0.999[3] | >0.999 | 0 (0.0%) | 0 (0.0%) | 2 (0.9%) | 10 (1.8%) | 0.648[3] | 0.729 | 0 (0.0%) | 8 (1.6%) | 4 (1.2%) | 0.739[3] | 0.832 |
| Herbal medicine | 24 (2.7%) | 0 (0.0%) | 24 (3.1%) | 0 (0.0%) | 0.190[3] | 0.671 | 2 (10.0%) | 2 (2.4%) | 6 (2.8%) | 14 (2.5%) | 0.230[3] | 0.375 | 0 (0.0%) | 12 (2.5%) | 12 (3.6%) | 0.221[3] | 0.393 |
| Physiotherapy | 10 (1.1%) | 0 (0.0%) | 9 (1.2%) | 1 (1.2%) | >0.999[3] | >0.999 | 0 (0.0%) | 0 (0.0%) | 2 (0.9%) | 8 (1.4%) | 0.756[3] | 0.756 | 0 (0.0%) | 10 (2.1%) | 0 (0.0%) | 0.013[3] | 0.092 |

[1]n (%)

[2]False discovery rate correction for multiple testing

[3]Fisher's exact test

[4]Pearson's Chi-squared test
